# Epidemiology of birth defects in teenage pregnancies: Based on provincial surveillance system in eastern China

**DOI:** 10.3389/fpubh.2022.1008028

**Published:** 2022-12-06

**Authors:** Xinning Chen, Haifeng Lou, Lijin Chen, Marie Parfaite Uwimana Muhuza, Danqing Chen, Xiaohui Zhang

**Affiliations:** ^1^Department of Obstetric, Women's Hospital, Zhejiang University School of Medicine, Hangzhou, China; ^2^School of Medicine, Zhejiang University, Hangzhou, China; ^3^Public Health, Zhejiang University, Hangzhou, China; ^4^Department of Women's Health, Women's Hospital, Zhejiang University School of Medicine, Hangzhou, China

**Keywords:** birth defects, congenital anomalies, adolescent mothers, teenage pregnancy, gastroschisis, NTDs

## Abstract

**Background:**

Healthcare for adolescents and birth defects (BD) prevention are highlighted public health issues. The epidemiology of birth defects in teenage pregnancies has not been studied extensively.

**Objectives:**

To investigate the prevalence trend and spectrum of BDs among teenage mothers.

**Methods:**

This observational study covered all births registered in the BD surveillance system in Zhejiang Province, China, during 2012–2018. The annual change in the prevalence of BDs among adolescent mothers was estimated. Crude relative ratios using the BD categories in teenage pregnancies were calculated and compared with those in women aged 25–29 years.

**Results:**

Overall, 54,571 BD cases among 1,910,977 births were included in this study, resulting in an overall prevalence of 234.64 to 409.07 per 10,000 births from 2012 to 2018 (*P*_trend_ < 0.001) in total population. The prevalence of birth defects in teenage pregnancies increased from 247.19 to 387.73 per 10,000 births in 2012–2018 (*P*_trend_ = 0.024). The risks of neural tube defects (relative risk [RR] = 3.15, 95% confidence interval [CI] 2.56, 3.87), gastroschisis (RR = 7.02, 95% CI 5.09, 9.69), and multiple birth defects (RR=1.27, 95% CI 1.07, 1.52) were higher in teenage pregnancies than those in women aged 25–29 years.

**Conclusions:**

We found a distinctive spectrum of BDs, with higher proportions of fatal or multiple anomalies in infants born to teenage mothers than in those born to adults aged 25–29 years. These results emphasize the importance of providing adolescents with better access to reproductive and prenatal care.

## Introduction

Birth defects (BDs) are structural or functional malformations that occur during or before birth. BDs are the primary cause of stillbirths and infant deaths (1, 2). In 2016, approximately 3–6% infants were born with BDs, contributing to 0.3 billion associated newborn deaths and accounting for 11.3% of neonatal deaths globally (3). It has been widely reported that maternal age is strongly associated with congenital anomalies. Most previous studies have reported elevated risks of BDs, particularly chromosomal anomalies, in mothers of an advanced age (4–7).

Although the World Health Organization has reported a declining trend in adolescent pregnancies (8), the rates and absolute number of teenage mothers have remained high in many countries (9, 10). Every year, 21 million adolescent women (aged 15–19 years) in developing regions become pregnant (8). Compared to women aged 20–35 years, several studies have observed higher risks of overall BDs, and of some specific defects, such as hydrocephaly, anencephaly, omphalocele, gastroschisis, and polydactyly, in infants born to younger women (11–13). Most previous studies in this field were conducted several years ago or were single-center studies. Thus, there is no current comprehensive overview of the correlations between pregnancies in younger women and BDs.

China has also faced a high burden of BDs, with the population prevalence of BDs ranging from 4 to 6% (14). Fertility rate in women aged 15–19 years in China was 7.6/1,000 during 2015–2020, which was lower than the world rate of 46.7/1,000 (15). As China has the largest population in the world, it is necessary to strengthen adolescent health. However, studies on the occurrence of BDs in pregnancies of younger women in China are rare.

Thus, in this study, the epidemiology and risks of BDs in infants born to teenage women were investigated using data from a provincial hospital-based BD surveillance system in Zhejiang Province, a province in eastern China with a population of more than 57 million people. The results of this study can help to improve BD prevention measures and adolescent healthcare in China, as well as in other developing countries.

## Methods

### Study population and data source

We retrospectively retrieved all births registered in the hospital-based BD surveillance system in Zhejiang Province, China, from January 2012 to December 2018. This passive reporting system consists of 90 hospitals located in 30 regions in the province, which observe 30% of the annual births in this province, since the hospital delivery rate remains at 100% in Zhejiang Province. The surveillance hospitals are determined by stratified sampling according to the regional distribution and birth number, comprising tertiary-, district-, county-, and community-level facilities, which are all licensed to provide maternity and newborn services.

BD cases are confirmed by trained obstetricians and pediatricians in the surveillance hospitals. Serological screening, noninvasive prenatal testing (NIPT), ultrasound scanning, and magnetic resonance imaging are the main methods to diagnose BDs, while genetic analysis and autopsy are also offered when necessary. BDs are mainly diagnosed prenatally and ascertained at delivery, whereas some anomalies are diagnosed within 7 days after birth. The data are submitted to the Women's Hospital, Zhejiang University School of Medicine, using a web-based information system. Quality control of the BD surveillance system is strictly and routinely carried out by professional doctors in the surveillance hospitals, regional maternal and child centers, and the Women's Hospital, Zhejiang University School of Medicine. This involves the accuracy of BD identification, data integrity and misreporting of BD. The underreporting rates of the total number of BDs should be <1% (16).

### Exposures and outcomes

Our main exposure variable was the maternal age at delivery. Pregnancy in adolescent females below the age of 20 years was defined as teenage pregnancy. We compared the characteristics of adolescent mothers with those of mothers of other age groups. Among them, the 25–29-year old group was selected as the reference group, as this group is commonly referred as the appropriate reproductive age (17–19) with low adverse perinatal outcomes, and comprised the largest proportion in this study. The full data for all age groups are presented in Supplementary Table 1. The BD system also reported other exposure variables for cases of BDs, including the maternal education and place of residence, while it only reported the total number of women delivered by age group in each year for denominators (total births in these surveillance hospitals).

The primary outcome of this study was the BD type. The surveillance system reported 25 major BDs with obvious structural or functional defects which had surgical, medical or cosmetic importance (20), according to the International Classification of Diseases, 10th Revision (ICD-10, Q00–Q99) (21). Anencephaly, encephalocele and spina bifida were collectively referred to as neural tube defects (NTDs). For congenital heart defects (CHD), we excluded isolated patent foramen ovale and isolated patent ductus arteriosus (PDA) in preterm births, whereas we included atrial septal defect (ASD) ≥ 3 mm and PDA ≥ 3 mm. Furthermore, multiple BDs were defined as the concurrence of two or more structural or developmental abnormalities in the same fetus or newborn, affecting at least two organ systems. Thus, one case of multiple BDs could be assigned and counted in more than one BD subtype, however cases of single chromosomal abnormalities were excluded from multiple BDs. The secondary outcomes of this study included gestational age at delivery, infant sex and perinatal outcomes. Cases delivered before and after 20 weeks of gestation were both required to be reported, as the perinatal period was defined as commencing at 20 weeks of gestation (22). Perinatal outcomes included livebirth (including infants who survived or died within the first week of birth), spontaneous fetal loss (before or after 20 weeks of gestation) and elective terminations of pregnancy due to fetal anomalies (TOPFA) at any gestational week. Spontaneous fetal loss, TOPFA and newborn death within 7 days of birth were further categorized as poor perinatal outcomes.

### Statistical analysis

The characteristics of the mothers and offspring in the different maternal age groups were compared using the chi-square test or Fisher's exact test for categorical variables, as appropriate. The Cochran–Armitage test was used for trend analysis according to the annual changes and maternal age changes.

The prevalence of BDs was calculated as (the number of BDs among livebirths + spontaneous fetal loss + TOPFA)/ (the total number of births in the population) ^*^10,000. For each BD subtype in teenage pregnancies, the crude relative ratios (RRs) and 95% confidence intervals (CIs) were calculated relative to the 25–29-year age group, while stratified analysis by infant sex for each BD subtypes was conducted. To depict the different profiles of the BD subtypes in the maternal age group, the BD subtypes were sorted in descending order of prevalence. Statistical analysis was performed using SPSS (version 22.0; Chicago, IL, USA) and MedCalc Software version 15.0 (Ostend, Belgium). Graphs and charts were constructed using GraphPad Prism 8 (GraphPad, San Diego, CA, USA). Statistical significance was set at *P* < 0.05, and all p-values were two-tailed.

### Missing data

Missing data represented <1% of all the variables (maternal education, region, number of embryos, and infant sex) in patients with BD (Table 1). However, these characteristic variables were not available for denominators; therefore, further adjustment was not made when calculating the RRs of BDs for teenage pregnancies compared with the reference group.

**Table 1:** Maternal and their offspring's characteristics of total births with birth defects according to maternal age.

**Valuable**	**Age**<**20**		**Age 25–29**		**P**
	***N***	**%**	***N***	**%**	
**Total cases with BDs**	1,385		21,855		
**Maternal region (2 missing)**					<0.001
Urban	626	45.2	14,058	64.32	
Rural	759	54.8	7,795	35.67	
**Maternal education (12 missing)**					<0.001
Illiterate	15	1.08	117	0.54	
Primary school	258	18.63	1,503	6.88	
Junior high school	871	62.89	6,135	28.07	
Senior high school	179	12.92	4,186	19.15	
Undergraduate or higher	60	4.33	9,904	45.32	
**Singleton or multiple births (32 missing)**					0.009
Singleton	1,321	95.38	20,439	93.52	
Multiple births	63	4.62	1,385	6.34	
**Infant sex (70 missing)**					0.69
Male	772	55.74	11,929	54.58	
Female	571	41.23	9,268	42.41	
Unknown^a^	37	2.67	593	2.71	
**Perinatal outcomes (7 missing)**					<0.001
Livebirth^b^					
Survive within the first week	1,012	73.07	16,049	73.43	
Die within the first week	28	2.02	205	0.94	
Spontaneous fetal loss					
< 20 w	5	0.36	25	0.11	
≥20 w	31	2.24	230	1.05	
TOPFA^b^	309	22.31	5,338	24.42	
**Delivery time**					0.002
Delivered before 20 w	68	4.91	722	3.3	
Delivered after 20 w	1,317	95.09	21,133	96.7	
**Time of diagnosis (19 missing)**					0.31
Prenatal	406	29.31	6,702	30.67	
Postpartum	977	70.54	15,136	69.26	
**BD Types**					0.02
Isolated BD	1,255	90.61	20,177	92.32	
Multiple BD	130	9.49	1,678	7.68	

a Unknown indicates that infant sex could not be identified.

b Livebirth refers to an infant showing signs of life after birth, and it was further categorized as (1) an infant who survive within the first week, (2) an infant die within the first week. TOPFA includes TOPFA at any gestational age.

BD, birth defect; TOPFA, elective terminations of pregnancy due to fetal anomalies; w, weeks.

### Ethics approval

This study was approved by the Ethics Committee of the Women's Hospital, Zhejiang University School of Medicine (No. 2018KY036). The privacy of all participants was protected.

## Results

### Characteristics of births with BDs by maternal age

Between 2012 and 2018, there were 54,571 BD cases among 1,910,977 births, according to the BD surveillance system in Zhejiang Province, giving a BD prevalence of 234.64 to 409.07 per 10,000 births (*P*_trend_ < 0.001) in total population. Generally, 2.61% of the mothers (*n* = 49,910) in the entire denominator were younger than 20 years, 19.25% (*n* = 367,927) were aged 20–24 years, 42.91% (*n* = 819,948) were aged 25–29 years, 23.86% (*n* = 456,012) were aged 30–34 years, and 11.36% (*n* = 217,180) were older than 35 years. Teenage pregnancies accounted for 2.54% of all births involving BDs. Among these, 2.96% were mothers aged 12–15 years, and 97.04% were aged 16–19 years. The earliest gestational age for recorded spontaneous pregnancy loss and TOPFA was 11 gestational weeks.

**Figure 1:**
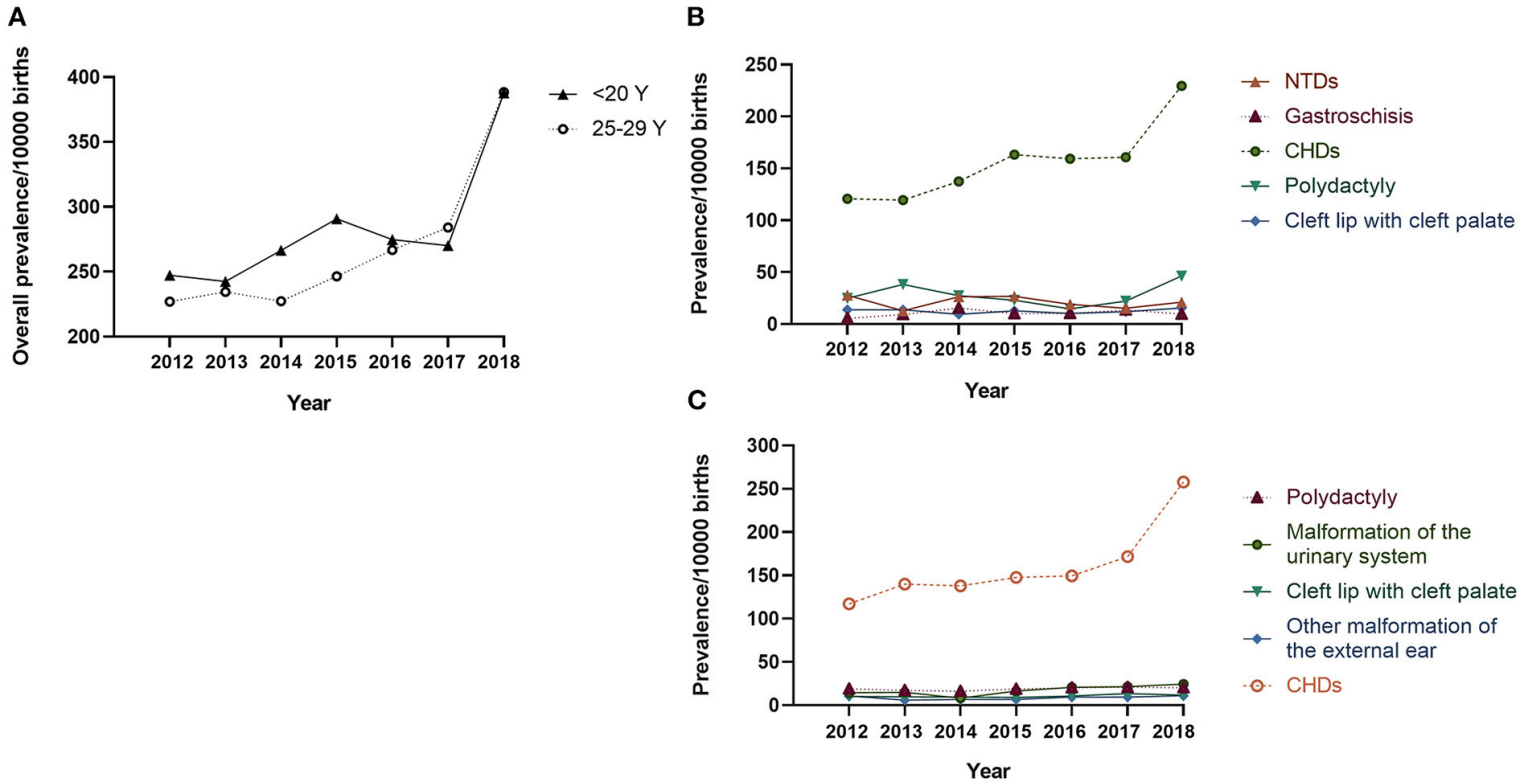
Prevalence of birth defects in teenage pregnancies per 10,000 births: 2012–2018 Legend: **(A)** The solid line indicates the prevalence of overall BDs in infants born to mothers < 20 years old, and the dotted line indicates the prevalence of overall BDs in infants born to mothers aged 25–29 years; **(B)** Prevalence of the top-five BD subtypes in infants born to mothers < 20 years old; **(C)** Prevalence of the top-five BD subtypes in infants born to mothers aged 25–29 years. CHD, congenital heart defect; NTD, neural tube defect.

The proportion of teenage pregnancies decreased significantly from 2013 to 2018 (3.23% −1.95%, *P*_trend_ < 0.001, Supplementary Figure 1). In contrast, the prevalence of BDs in teenage pregnancies increased from 247.19 to 387.73 per 10,000 births from 2012 to 2018 (*P*_trend_ = 0.024, Figure 1A), particularly from 2017 to 2018. Analysis of BD subtypes revealed that CHD was driving the increase, from 120.82 to 229.55 per 10,000 births (P_trend_ < 0.001, Figure 1B). A similar upward trend in overall BDs and CHD was also observed in infants born to women aged 25–29 years (Figure 1C).

The characteristics of the BD cases by maternal age are compared in Table 1 (full data with more maternal age groups are presented in Supplementary Table 1). Compared to the 25–29-year old group, teenage mothers were more likely to live in rural regions (*P* < 0.001), have primary-level education (*P* < 0.001), and have singleton pregnancies (*P* = 0.009). Teenage pregnancies were more often associated with multiple malformations (*P* = 0.02). The BDs associated with teenage gestation were more likely to suffer from a higher proportion of neonatal deaths within 7 days after delivery (2.02%).

### Distribution of specific BDs by maternal age

Table 2 shows the prevalence and ranking of the BD subtypes in the different age groups. The top five BD subtypes in teenage pregnancies were CHD, polydactyly, NTDs, cleft lip with cleft palate, and gastroschisis. Stratified analysis in teenage pregnancy showed no significant difference in the proportion of the top five BD subtypes among ≤15, 16–17, 18–19-year-old group, or between primipara and multipara (Supplementary Table 2). CHD, polydactyly, and cleft lip with cleft palate remained the top five BDs in the 25–29-years age group, while NTDs dropped to the eighth position, and gastroschisis dropped to the 21st position in the 25–29-years age group.

**Table 2:** Prevalence and ranking of 25 BD subtypes by maternal ages.

	**Age**<**20**			**Age 25–29**		
**BD subtypes**	**N**	**Prevalence /10,000 births**	**Rank**	**N**	**Prevalence/10,000 births**	**Rank**
CHD	755	151.27	1	13,060	159.28	1
Polydactyly	139	27.85	2	1,541	18.79	2
NTDs	107	21.44	3	559	6.81	8
spina bifida	52	10.42		271	3.31	
anencephaly	33	6.61		192	2.34	
encephalocele	22	4.41		96	1.17	
Cleft lip with cleft palate	62	12.42	4	866	10.56	4
Gastroschisis	53	10.62	5	124	1.51	21
Malformation of the urinary system	49	9.82	6	1,408	17.17	3
Other malformation of the external ear	48	9.62	7	689	8.4	5
Cleft lip without cleft palate	42	8.41	8	395	4.82	13
Other unclassified malformation	42	8.41	9	656	8	6
Syndactyly	35	7.01	10	575	7.01	7
Clubfoot	32	6.41	11	558	6.81	9
Congenital hydrocephalus	31	6.21	12	426	5.19	11
Hypospadias	28	5.61	13	507	6.18	10
Atresia of the rectum and anus	19	3.81	14	249	3.04	18
Limb reduction defects	18	3.61	15	310	3.78	15
Microtia	13	2.6	16	269	3.28	17
Cleft palate without cleft lip	12	2.4	17	281	3.42	16
Omphalocele	11	2.2	18	195	2.38	19
Trisomy 21 syndrome	11	2.2	19	380	4.63	14
Other chromosomal abnormalities	8	1.6	20	400	4.88	12
Diaphragmatic hernia	6	1.2	21	146	1.78	20
Congenital esophageal atresia	5	1	22	66	0.8	22
Exstrophy of the urinary bladder	0	0	23	5	0.06	23

Gray indicates NTDs.

BD, birth defect; CHD, congenital heart defect; NTD, neural tube defect.

### Risks of BDs in teenage mothers according to BD subtype

**Figure 2:**
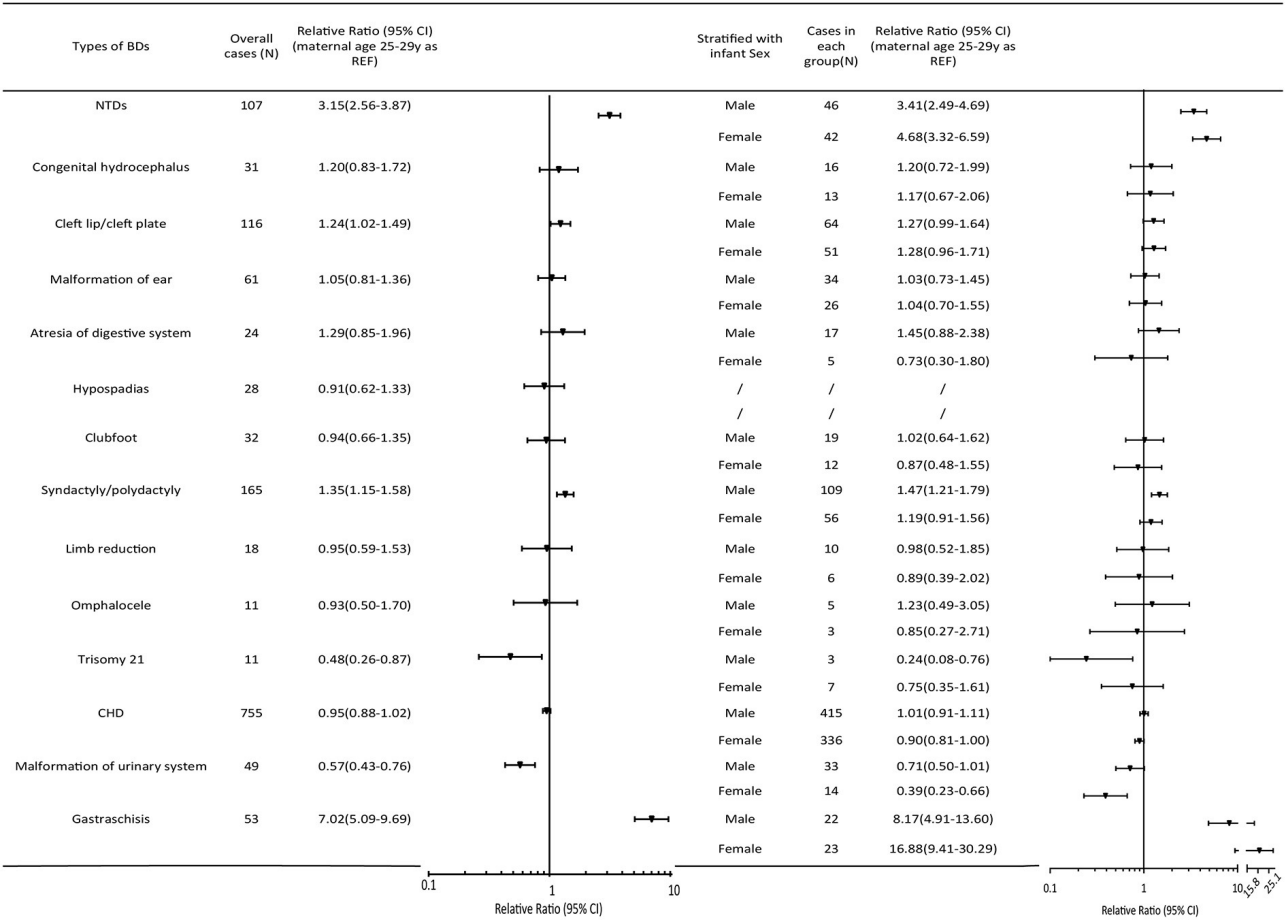
Relative ratios (95%CI) of BD subtypes in the maternal age group < 20 years relative to the 25–29-year-old group. The pooled relative ratio and infant sex-specific relative ratio are expressed in comparison to those of infants born to mothers aged 25–29 years. Cleft lip/palate comprises cleft lip, cleft palate, and cleft lip with cleft palate. NTDs include spina bifida, anencephaly, and encephalocele. Atresia of the digestive system includes atresia of the rectum, anus, and esophagus. Diaphragmatic hernia, exstrophy of the urinary bladder, and other chromosomal abnormalities (except trisomy 21) were not presented in the Figure because of the low prevalence. CHD, congenital heart defect; CI, confidence interval; NTD, neural tube defect.

No significant relationship was observed between teenage pregnancy and an increased risk of BDs overall (RR = 1.04, 95% CI 0.99, 1.10). However, teenage pregnancy was significantly associated with an increased risk of multiple BDs (RR=1.27, 95% CI 1.07, 1.52). As for the different BD subtypes (Figure 2), teenage mothers were observed to have three times higher risk of having a baby with NTDs (RR = 3.15, 95% CI 2.56, 3.87), and seven times higher risk of having a baby with gastroschisis (RR = 7.02, 95% CI 5.09, 9.69) compared with mothers aged 25–29 years. In contrast, an age < 20 years was a protective factor for trisomy 21 (RR = 0.48, 95% CI 0.26, 0.87), other chromosomal BDs (RR = 0.33, 95% CI 0.16, 0.66), and urinary malformations (RR = 0.57, 95% CI 0.43, 0.76). The results were consistent when stratified according to infant sex.

### Perinatal outcomes of BDs in teenage pregnancies

In total, 373 cases of teenage pregnancies with BDs had poor perinatal outcomes, including 309 cases terminated, 36 cases involving spontaneous fetal loss, and 28 cases involving neonatal deaths within 7 days after delivery. When we analyzed the composition of teenage pregnancy-associated BDs with poor perinatal outcomes (Supplementary Figure 2), we found that 313 and 60 cases suffered from isolated BD and multiple BDs, respectively. In cases of an isolated BD, central nervous system anomalies (*N* = 97, 26.00%), CHD (*N* = 65, 17.43%), and integumental anomalies (*N* = 49, 13.14%) were the three major causes of a poor prognosis. Notably, for the central nervous system, the risk was primarily attributable to spina bifida (*N* = 42).

## Discussion

The results of this study demonstrate a marked increase in the prevalence of BDs in teenage pregnancies during 2012–2018 in eastern China. Teenage mothers who delivered BDs were more likely to live in rural areas and have a primary level of education than women aged 25–29 years. In terms of BD subtypes, CHD is the predominant anomaly in teenage pregnancies. Nevertheless, when compared to the 25–29-year- old group, young maternal age (< 20 years) was associated with an elevated risk of NTDs and gastroschisis.

### Interpretation

We found an overall decline in the proportion of teenage mothers in recent years, consistent with the trend in USA during 1978–2012 (23, 24). The proportion reached at approximately 2% in 2018, similar to that (4%) in the USA in 2018 (25), which indicates a marked achievement in China, attributable to the close collaboration of the government, public health organizations, medical workers, and schools (26). Compared to the 25–29-year-old group, teenage mothers comprised a larger proportion residing in rural areas and with illiteracy or primary education. However, young women may have access to a higher level of education for the rest of their lives, and this difference should be interpreted with caution. It is undeniable that education is still a protective factor against teenage maternity (27). Strengthening sexual and reproductive healthcare education among adolescents is a worldwide challenge, which underlines the priority of school-based education and community-based programs (28, 29). Despite free universal access to primary and secondary education provided by the Chinese government, educational attainment also reflects socio-economic wellbeing. Zhejiang Province is a relatively economically developed province in China, nevertheless, eliminating teenage maternity remains a challenge.

The upward trend in the overall prevalence of BDs in teenage pregnancies warrants further continuous attention. Our subtype analysis showed that CHD was driving the pattern of increase, which is in line with the global increase trend in CHD (30, 31). However, the prevalence of CHD in our study was much higher than the total prevalence of 93.42 per 10,000 in Asia reported by a systematic review (30). This disparity was likely due to the wide utilization of echocardiography in the Zhejiang Province, as well as the inclusion of a large proportion of ASD and PDA cases in this study. Notably, the sharp increase in CHD prevalence and overall BD prevalence, particularly between 2017 and 2018, could be explained by a standardized strategy in neonatal CHD screening and NIPT screening proposed by the National Center for Women and Children's Health in China in 2017, as a consistent upward trend was also observed in infants born to mothers aged 25–29 years. Although many studies have indicated an increasing trend for gastroschisis among adolescent mothers (32–34), our study observed a rising trend only during 2012–2014, possibly because of the different observation periods.

Teenage pregnancy is associated with a higher risk of certain types of BDs (11, 13, 35, 36). Our study reported a nearly three-fold higher risk of NTDs associated with teenage pregnancy. A recent study by Liu et al. further illustrated that spina bifida accounted for the largest portion of the risk (17), supporting our findings (Table 2). The elevated risk of NTDs may be attributed to the unawareness of the benefits of folic acid (17) or vitamin supplementation in adolescent mothers (37) in unplanned pregnancy. Notably, approximately 10% of teenage mothers were multiparous. Some teenage mothers, especially those aged 18–19 years, had repeated pregnancies and remained at risk of delivering offspring with NTDs (Supplementary Table 2), even in their second pregnancies. This suggests that these teenage mothers may be at risks of repeated exposure to an unhealthy lifestyle or the same supplement. This has prompted the importance of reproductive health education in adolescents. Exposure information such as maternal drug or supplementation use, particularly the use of folic acid, was lacking in our study. It was unclear whether the folic acid use or the low maternal age itself had a greater impact on the high risk of NTDs. This needs to be further confirmed by studies with more adequate data in the future.

As for gastroschisis, observational studies from Mexico and the USA have indicated an prevalence of approximately 10 per 10,000 live births among adolescent mothers, which was markedly higher than the prevalence in the ≥ 20-year age group (38, 39), equivalent to the prevalence in our study. Poor nutrition and unhealthy lifestyle behaviors, such as smoking or alcohol intake, might partially explain the high prevalence of gastroschisis (40). Additionally, in accordance with the findings of previous researches, multiple BDs occurred more frequently in teenage pregnancies than in pregnancies involving other age groups (6). Possibly elevated germline mutation rates in adolescent fathers (41) and teratogen exposure during an unintended pregnancy have played a role. Correspondingly, the higher risk of poor neonatal outcomes in the adolescent pregnancies might be interpreted by the large proportion of severe and multiple abnormalities in teenage pregnancies and a slightly lower TOPFA proportion. It is noteworthy that most of these severe malformations can be identified prenatally (42), and may be terminated after an ethical review. This finding demonstrates the necessity of providing education to adolescents and expanding prenatal visit coverage.

### Strengths and limitations

The primary strength of this study is the large sample size and full coverage of the BD spectrum in young women. To our knowledge, few studies have presented a complete ranking of BD subtypes in teenage pregnancies. Moreover, we focused on all births with BDs, including early fetal loss. This provides a more comprehensive understanding of the association between perinatal outcomes and maternal age.

This study also has several limitations. First and foremost, we were unable to control for some confounding factors. Demographic data, such as living area and education level, were not obtained for denominators without BDs. Thus, the adjusted RRs of BDs for teenage pregnancy, could not be calculated after considering all of the confounding factors. Second, it was difficult to capture all early fetal losses within 12 gestational weeks. Only information on early neonatal death within 7 days after delivery, was included. This may have led to an underestimation of the anomalies. In future studies, the long-term neonatal or pediatric prognosis of the children born to teenage mothers should be investigated.

## Conclusions

In conclusion, the prevalence of BDs in teenage pregnancies has increased during the period of 2012–2018 in China. Teenage pregnancy is associated with a higher risk of certain types of BDs, particularly NTDs, gastroschisis, and multiple BDs. The growing prevalence of BDs and distinctive spectrum of BD in teenage pregnancy highlight the significance of appropriate reproductive education programs and intensive healthcare services for adolescents.

## Data availability statement

The data cannot be shared publicly because we are obligated to protect the privacy of participants within the BD surveillance system of Zhejiang Province, China. Data are available for researchers who meet the criteria for access to confidential data.

## Ethics statement

The studies involving human participants were reviewed and approved by Women's Hospital, Zhejiang University School of Medicine. Written informed consent to participate in this study was provided by the participants' legal guardian/next of kin.

## Author contributions

XC: conceptualization, data curation, data analysis, interpretation, writing, and review and editing. HL: literature search, data interpretation, and review and editing. LC: data analysis, data interpretation, and review and editing. MM: literature search and review and editing. DC: study design, data collection, and review and editing. XZ: conceptualization, study design, data collection, writing, and review and editing. All authors contributed to the article and approved the submitted version.

## Conflict of interest

The authors declare that the research was conducted in the absence of any commercial or financial relationships that could be construed as a potential conflict of interest.

## Publisher's note

All claims expressed in this article are solely those of the authors and do not necessarily represent those of their affiliated organizations, or those of the publisher, the editors and the reviewers. Any product that may be evaluated in this article, or claim that may be made by its manufacturer, is not guaranteed or endorsed by the publisher.
