# Supplementary figures and images for: Epidemiology of birth defects in teenage pregnancies: Based on provincial surveillance system in eastern China
Source: Front Public Health. 2022 Dec 6;10:1008028. doi: 10.3389/fpubh.2022.1008028 (PMC9763884; doi:10.3389/fpubh.2022.1008028)

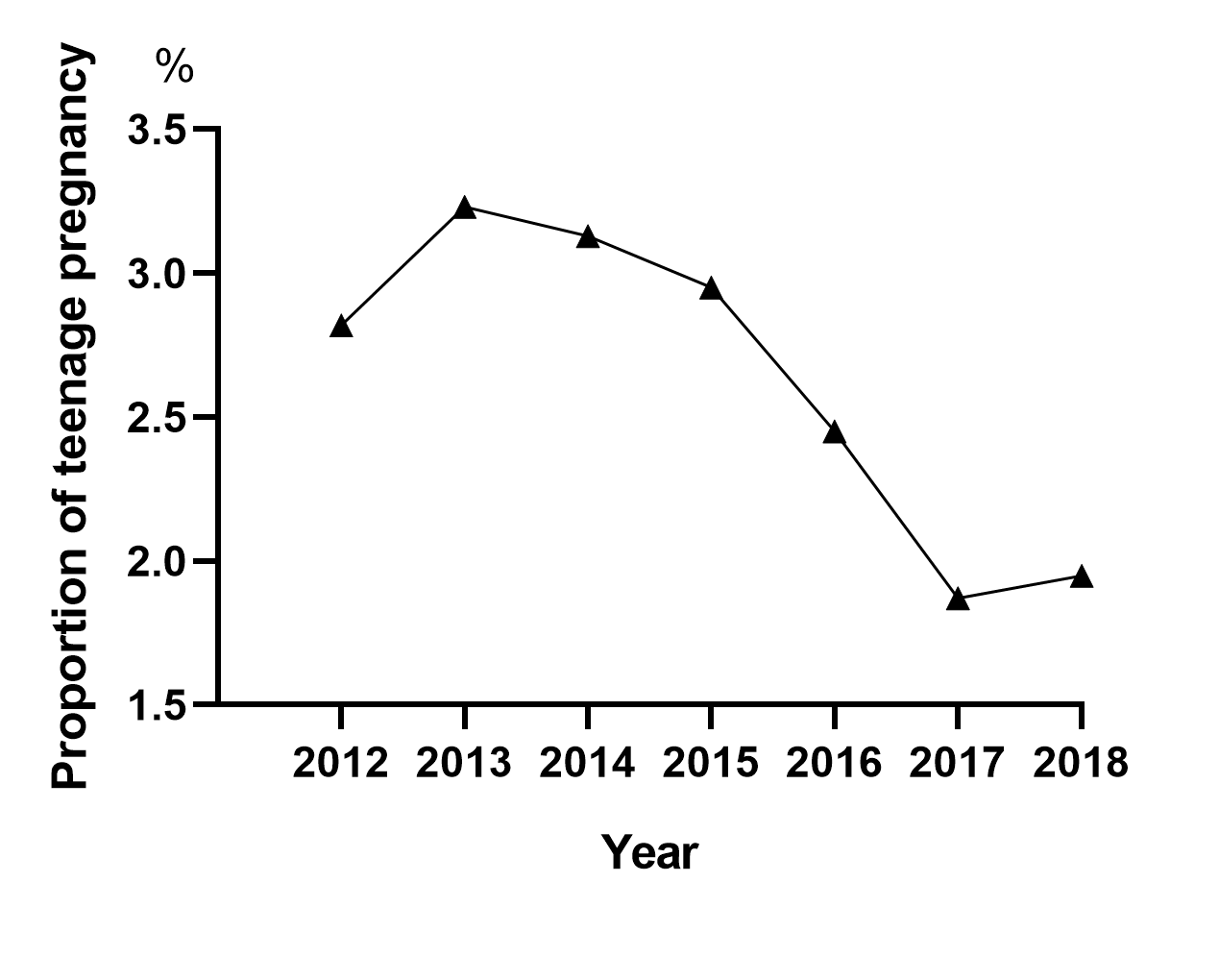

Supplement: Supplementary file 1 [file Image_1.tif]

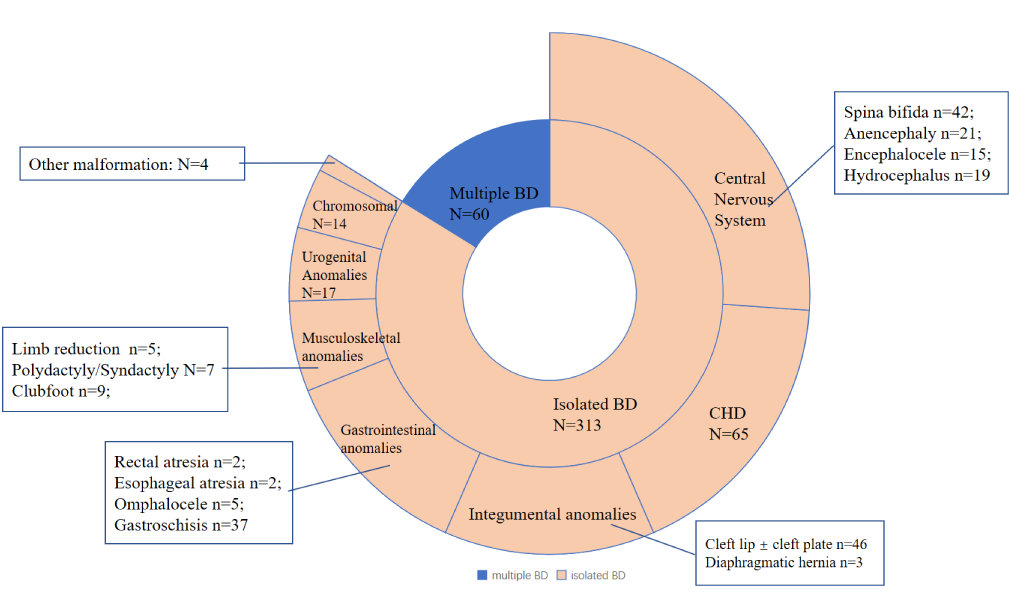

Supplement: Supplementary file 2 [file Image_2.TIF]
