# Supplementary material for: Epidemiology of birth defects in teenage pregnancies: Based on provincial surveillance system in eastern China
Source: Front Public Health. 2022 Dec 6;10:1008028. doi: 10.3389/fpubh.2022.1008028 (PMC9763884; doi:10.3389/fpubh.2022.1008028)
Supplement: Supplementary file 3 [file Table_1.docx]

Table 1 Maternal and their offspring’s characteristics of total births with birth defects according to maternal age

| Valuable | Age<20 | | Age 20-24 | | Age 25-29 | | Age 30-34 | | | Age ≥35 | | P value |
| --- | --- | --- | --- | --- | --- | --- | --- | --- | --- | --- | --- | --- |
|  | N | % | N | % | N | % | | N | % | N | % |  |
| **Total cases** | 1385 |  | 9706 |  | 21855 |  | | 13365 |  | 8260 |  |  |
| **Maternal Region (5 missing)** | | |  |  |  |  | |  |  |  |  | <0.001* |
| Urban | 626 | 45.20 | 4966 | 51.16 | 14058 | 64.32 | | 9056 | 67.76 | 5382 | 65.16 |  |
| Rural | 759 | 54.80 | 4740 | 49.84 | 7795 | 35.67 | | 4306 | 32.22 | 2878 | 34.84 |  |
| **Maternal Education (32 missing)** | | |  |  |  |  | |  |  |  |  | <0.001 |
| illiterate | 15 | 1.08 | 68 | 0.70 | 117 | 0.54 | | 100 | 0.75 | 159 | 1.92 |  |
| primary school | 258 | 18.63 | 969 | 9.98 | 1503 | 6.88 | | 1042 | 7.80 | 1087 | 13.16 |  |
| junior high school | 871 | 62.89 | 3847 | 39.64 | 6135 | 28.07 | | 4023 | 30.10 | 2669 | 32.31 |  |
| senior high school | 179 | 12.92 | 2418 | 24.91 | 4186 | 19.15 | | 2564 | 19.18 | 1516 | 18.35 |  |
| undergraduate or higher | 60 | 4.33 | 2398 | 24.71 | 9904 | 45.32 | | 5625 | 42.09 | 2826 | 34.21 |  |
| **Singleton or multiple births (62 missing)** | | |  |  |  |  | |  |  |  |  | 0.001* |
| singleton | 1321 | 95.38 | 9216 | 94.95 | 20439 | 93.52 | | 12345 | 92.37 | 7759 | 93.93 |  |
| multiple births | 63 | 4.62 | 480 | 4.95 | 1385 | 6.34 | | 1009 | 7.55 | 492 | 5.96 |  |
| **Infant Sex (167 missing)** |  |  |  |  |  |  | |  |  |  |  | 0.18 |
| Male | 772 | 55.74 | 5301 | 54.62 | 11929 | 54.58 | | 7393 | 55.32 | 4458 | 53.97 |  |
| Female | 571 | 41.23 | 4100 | 42.24 | 9268 | 42.41 | | 5584 | 41.78 | 3501 | 42.38 |  |
| Unknown | 37 | 2.67 | 285 | 2.94 | 593 | 2.71 | | 347 | 2.60 | 265 | 3.21 |  |
| **Perinatal outcomes (27 missing)** | |  |  |  |  |  | |  |  |  |  | <0.001 |
| Livebirth b |  |  |  |  |  |  | |  |  |  |  |  |
| Survive within the first week | 1012 | 73.07 | 6954 | 71.65 | 16049 | 73.43 | | 9782 | 73.19 | 5646 | 68.35 |  |
| Die within the first week | 28 | 2.02 | 130 | 1.34 | 205 | 0.94 | | 120 | 0.90 | 69 | 0.84 |  |
| Spontaneous fetal loss |  |  |  |  |  |  | |  |  |  |  |  |
| <20w | 5 | 0.36 | 16 | 0.16 | 25 | 0.11 | | 17 | 0.13 | 24 | 0.29 |  |
| ≥20w | 31 | 2.24 | 154 | 1.59 | 230 | 1.05 | | 178 | 1.33 | 115 | 1.39 |  |
| TOPFA b | 309 | 22.31 | 2444 | 25.18 | 5338 | 24.42 | | 3263 | 24.41 | 2400 | 29.06 |  |
| **Delivery time (3 missing)** | | |  |  |  |  | |  |  |  |  | 0.12* |
| Delivered before 20 w | 68 | 4.91 | 367 | 3.78 | 722 | 3.30 | | 417 | 3.12 | 288 | 3.49 |  |
| Delivered after 20 w | 1317 | 95.09 | 9336 | 96.20 | 21133 | 96.70 | | 12948 | 96.88 | 7972 | 96.51 |  |
| **Time of diagnosis (40 missing)** |  |  |  |  |  |  | |  |  |  |  | <0.001* |
| Prenatal | 406 | 29.31 | 3112 | 32.06 | 6702 | 30.67 | | 4089 | 30.59 | 2952 | 35.74 |  |
| Postpartum | 977 | 70.54 | 6588 | 67.88 | 15136 | 69.26 | | 9266 | 69.33 | 5303 | 64.20 |  |
| **BD Types** |  |  |  |  |  |  | |  |  |  |  | 0.23* |
| isolated BD | 1255 | 90.61 | 8957 | 92.28 | 20177 | 92.32 | | 12315 | 92.14 | 7529 | 91.15 |  |
| multiple BD | 130 | 9.49 | 749 | 7.72 | 1678 | 7.68 | | 1050 | 7.86 | 731 | 8.85 |  |

*P* values were derived from chi-squared tests. *P** was derived from Cochran-Armitage trend test

1. Unknown indicates that infant sex could not be identified; no information indicates missing data on infant sex
2. Livebirth refers to an infant showing signs of life after birth, and it was further categorized as (1) an infant who survive within the first week, (2) an infant die within the first week. TOPFA includes TOPFA at any gestational age.

Abbreviations: BD, birth defect; TOPFA, elective terminations of pregnancy due to fetal anomalies; w, weeks

sTable 2 Birth defects born to teenage mothers with different maternal age and parity

|  | Maternal age | | | | | | Parity | | | |
| --- | --- | --- | --- | --- | --- | --- | --- | --- | --- | --- |
|  | ≤15y |  | 16-17y |  | 18-19y |  | Primipara |  | Multipara |  |
|  | N | % | N | % | N | % | N | % | N | % |
|  | 41 |  | 323 |  | 1021 |  | 1236 |  | 141 |  |
| **Perinatal outcomes** **^a^** |  |  |  |  |  |  |  |  |  |  |
| Live birth |  |  |  |  |  |  |  |  |  |  |
| Survive within the first week | 28 | 68.3 | 235 | 72.7 | 748 | 73.3 | 888 | 71.9 | 116 | 82.3 |
| Die within the first week | 0 | 0 | 5 | 1.6 | 23 | 2.3 | 23 | 1.9 | 5 | 3.5 |
| Spontaneous fetal loss |  |  |  |  |  |  |  |  |  |  |
| <20w | 1 | 2.4 | 0 | 0 | 5 | 0.5 | 6 | 0.5 | 0 | 0 |
| ≥20w | 3 | 7.3 | 11 | 3.4 | 17 | 1.7 | 28 | 2.3 | 3 | 2.1 |
| TOPFA | 9 | 22 | 71 | 22.0 | 228 | 22.3 | 290 | 23.5 | 17 | 12.1 |
| **BD Types** |  |  |  |  |  |  |  |  |  |  |
| Isolated | 37 | 90.2 | 289 | 89.5 | 913 | 89.4 | 1103 | 89.2 | 129 | 91.5 |
| multiple | 4 | 9.8 | 34 | 10.5 | 108 | 10.6 | 133 | 10.8 | 12 | 8.5 |
| **Subtypes ^b^** |  |  |  |  |  |  |  |  |  |  |
| Gastroschisis | 4 | 9.8 | 14 | 4.3 | 35 | 3.4 | 46 | 3.7 | 7 | 5 |
| CHD | 17 | 41.5 | 178 | 55.1 | 560 | 54.8 | 667 | 54 | 60 | 42.6 |
| NTD | 3 | 7.3 | 34 | 10.5 | 70 | 6.9 | 100 | 8.1 | 7 | 5 |
| Polydactyly | 3 | 7.3 | 31 | 9.6 | 105 | 10.3 | 123 | 10 | 16 | 11.3 |
| Cleft lip with cleft palate | 3 | 7.3 | 12 | 3.7 | 47 | 4.6 | 55 | 4.4 | 7 | 5 |

1. Livebirth refers to an infant showing signs of life after birth, and it was further categorized as (1) an infant who survive within the first week, (2) an infant die within the first week. TOPFA includes TOPFA at any gestational age.
2. Top-five birth defects subtypes from teenage pregnancies were selected and compared according to maternal age and parity.

Abbreviations: BD, birth defect; TOPFA, elective terminations of pregnancy due to fetal anomalies; w, weeks
